# Long-Term Validation of a Consumer- vs. Industrial-Grade Indoor Radon and Air Quality Monitor at a School in Utah

**DOI:** 10.3390/s26165230

**Published:** 2026-08-18

**Authors:** John D. Beard, Maren M. Ball, Christopher Kitras, Philip B. Lundrigan

**Affiliations:** 1 Department of Public Health, Brigham Young University, Provo, UT 84602, USA; 2 Department of Electrical and Computer Engineering, Brigham Young University, Provo, UT 84602, USAlundrigan@byu.edu (P.B.L.)

**Keywords:** carbon dioxide, barometric pressure, indoor air quality, monitor, radon, relative humidity, school, temperature, validation, volatile organic compounds

## Abstract

In recent years, the demand for indoor radon and air quality monitors has increased while more affordable consumer-grade monitors have become available. The aim of our study was to assess the validity of the less expensive, non-calibrated, consumer-grade Lüft (*Sun*RADON LLC, Melbourne, FL, USA) compared to the more expensive, annually and manufacturer calibrated, industrial-grade Model 1028 XP (*Sun*RADON LLC, Melbourne, FL, USA). We collected data about 24 h moving averages of barometric pressure, carbon dioxide (CO_2_), radon, relative humidity, temperature, and volatile organic compounds (VOC) for approximately 29 weeks in 2024 in each of six rooms in a school in Utah. The mean difference in the co-located Lüfts’ and Model 1028 XPs’ 24 h moving average radon was −0.05 pCi/L of air (95% confidence interval: −0.24, 0.14, *p* = 0.50) and the concordance correlation coefficient (CCC) was 0.92. The Lüfts’ and Model 1028 XPs’ 24 h moving averages were similar for barometric pressure (CCC: 0.87), but the Lüfts’ 24 h moving averages were lower than the Model 1028 XPs’ for CO_2_, relative humidity, and VOC and higher for temperature. The Lüft provided valid measurements of 24 h moving averages for barometric pressure and radon, but not for CO_2_, relative humidity, temperature, and VOC.

## 1. Introduction

Radon is a noble gas found in four naturally occurring isotopes with the most stable isotope, ^222^Rn, posing the greatest risk to human health [1]. Approximately 40% of human radiation exposure is from ^222^Rn. ^222^Rn is a product of uranium decay, typically in soil, and has the longest half-life of the naturally occurring radon isotopes: approximately 3.82 days. As it decays, ^222^Rn emits radioactive alpha particles that can mix with aerosols and be inhaled by humans. Indoor radon exposure is a major health risk because radon gas can travel from soil up through cracks in building foundations and walls and get trapped inside [2]. Humans can also experience radon poisoning through ingestion of contaminated groundwater. Exposure to ^222^Rn is the leading risk factor for lung cancer among non-smokers and is the second leading risk factor for lung cancer among smokers [1,3]. The U.S. Environmental Protection Agency (EPA) recommends that mitigation measures be implemented if radon concentrations in a building are greater than or equal to 4.0 picocuries per liter (pCi/L) of air [4]. Due to increased awareness of indoor radon exposure in recent years, the demand for indoor radon monitors has increased and more affordable consumer-grade models have become available [5].

Some consumer-grade radon monitors also measure other air quality parameters, such as carbon dioxide (CO_2_), volatile organic compounds (VOCs), barometric pressure, relative humidity, and temperature. Like radon, each of these environmental exposures can have significant impacts on human health, even, and sometimes especially, in indoor settings. Indoor CO_2_ concentrations are often significantly higher than outdoor concentrations due largely to combustion reactions from household cooking, heating, and smoking, and exhalation of people [6,7]. Elevated CO_2_ concentrations can lead to respiratory issues and cognitive performance degradation. The primary sources of indoor VOCs are also indoor combustion reactions (cooking, heating, and smoking), as well as household building materials such as paint, flooring, and synthetic wooden furniture [8]. In schools, VOC exposures can occur from the use of adhesives, markers, paints, or cleaners [7]. Exposure to indoor VOCs, such as benzene, toluene, xylenes, and aldehydes, is associated with respiratory issues and several types of cancer [8]. Barometric pressure is known to be associated with a variety of health problems, including hypertension, myocardial infarction, stroke, migraine, epileptic seizures, and sensory disorders [9]. Relative humidity outside the optimal range is associated with negative health effects because it contributes to environments in which infectious disease transmission and respiratory disease symptoms can be exacerbated [10]. Extreme temperatures, both cold and hot, are associated with increased mortality through a variety of causes [11]. Consumer-grade radon monitors that measure additional air quality parameters can help consumers track a variety of potential indoor health risks.

The aim of our study was to assess the validity of the consumer-grade Lüft indoor radon and air quality monitor (*Sun*RADON LLC, Melbourne, FL, USA) compared to the industrial-grade Model 1028 XP indoor radon and air quality monitor (*Sun*RADON LLC, Melbourne, FL, USA). We chose the Model 1028 XP as the gold standard because it is an annually and manufacturer calibrated, industrial-grade monitor that reports hourly averages once every hour, whereas the Lüft is a non-calibrated, consumer-grade monitor that only reports 24 h moving averages once every hour. There have been a few previous studies that have assessed the validity of different indoor radon monitors for measuring radon concentrations, but no studies have compared the two monitors investigated here. Two studies assessed the validity of consumer-grade electronic models available to the public for measuring radon concentrations, including the Lüft, for one, two, and/or three weeks in laboratory environments [12,13]. Both studies found the Lüft monitor met established performance standards, but tended to slightly underestimate radon concentrations, when compared to an industrial-grade reference monitor. No prior studies assessed the validity of the Lüft for measuring radon concentrations compared to the Model 1028 XP, for longer than three weeks, or in a non-laboratory or field environment. In addition, no prior studies assessed the validity of the Lüft for measuring other indoor air quality parameters. For our study, we collected data regarding radon and other air quality parameters for approximately 29 weeks in 2024 in each of six rooms in a school in Utah and compared repeated measurements over time for the Lüft vs. the Model 1028 XP.

## 2. Materials and Methods

### 2.1. Study Design

We conducted a validation study from 12 January to 6 June and from 20 September to 15 November 2024. In Kitras et al. [14], we had the opportunity to deploy continuous radon monitors (CRMs) for an extended period of time in 2024 across many heating, ventilation, and air conditioning (HVAC) system zones in a school in Utah. We divided the study into four seasonal periods with three distinct phases within each seasonal period to measure the impact of different types of HVAC system schedules on radon and indoor air quality. Previous studies [15] and initial radon tests that we conducted in each room of the school using charcoal-based, short-term radon test kits (Alpha Energy Laboratories, Carrollton, TX, USA) for greater than or equal to 48 h from 30 January to 1 February 2023 affirmed that large structures do not have uniform radon distributions. In fact, five rooms in the school had radon concentrations greater than or equal to the EPA’s recommended action level of 4.0 pCi/L of air [4] during initial testing. Therefore, we used multiple CRMs to test six rooms (i.e., the five rooms that had radon concentrations greater than or equal to 4.0 pCi/L of air and one room that did not) in the school for the current validation study. In each indicated room, we co-located a Lüft and a Model 1028 XP within a few inches of each other on filing cabinets, counters, desks, or tables within approximately 2.5–4.5 feet (0.76–1.37 m) of the floor to determine whether the Lüft gave valid measurements of barometric pressure, CO_2_, radon, relative humidity, temperature, and VOC relative to the Model 1028 XP. We plugged each Lüft and Model 1028 XP into AC electrical power outlets for the duration of the study. Our study was determined by the Institutional Review Board at Brigham Young University not to be human subjects research.

### 2.2. Instruments

The Model 1028 XP is certified by the National Radon Proficiency Program, National Radon Safety Board, and Canadian National Radon Proficiency Program and is meant to be used by building and home inspectors [16]. The Model 1028 XP also has an Environmental Module that can be installed, which we did for our study, which allows the Model 1028 XP to record measurements about relative humidity, temperature, VOCs, barometric pressure, and CO_2_ in addition to radon (Table 1). VOCs detected included alcohols, aldehydes, aliphatic hydrocarbons, amines, aromatic hydrocarbons, ketones, and organic acids. Each Model 1028 XP cost $995.00 USD, and each Environmental Module cost $199.00 USD at the time of purchase. The Model 1028 XPs were calibrated for radon, but not the other indoor air quality parameters, by the manufacturer before we used them for our study [17]. The Model 1028 XPs performed self-baselining and thereby provided relative measures of VOCs and CO_2_, but absolute measures of relative humidity, temperature, barometric pressure, and radon. The measurements were logged every hour. Approximately once every two weeks, we retrieved the measurements from the Model 1028 XPs located in the school manually using OneRadon version 2.3.12 (*Sun*RADON LLC, Melbourne, FL, USA) [16], but the Model 1028 XP also has the option to add a Long-Term Evolution (i.e., cellular) module that allows the measurements to be retrieved remotely.

We selected the Lüft for this study due to its focus on ease of deployment, low cost ($249 USD each when we purchased them), and user-focused interface to retrieve data remotely via the OneRadon mobile app or cloud dashboard [18]. The Lüft records measurements about radon, relative humidity, temperature, VOCs, barometric pressure, and CO_2_ (Table 1). The Lüfts detected the same VOCs as the Model 1028 XPs [17]. The Lüfts also used the same hardware and approach (i.e., self-baselining) for measuring VOCs and CO_2_ as the Model 1028 XPs and thereby provided relative measures of VOCs and CO_2_, but absolute measures of relative humidity, temperature, barometric pressure, and radon. The measurements were logged every hour and were an average of the last 24 h (i.e., 24 h moving averages).

### 2.3. Statistical Analyses

We completed all data management and analyses using SAS version 9.4 (SAS Institute, Inc., Cary, NC, USA). The Lüfts logged 24 h moving averages once every hour. However, Model 1028 XPs logged hourly averages once every hour. Therefore, we averaged the Model 1028 XPs’ hourly averages to obtain 24 h moving averages so that the data collected by the Lüfts and Model 1028 XPs could be compared directly. We created dichotomous variables (Yes, No) for both the Lüfts’ and Model 1028 XPs’ radon concentrations to indicate whether or not the 24 h moving average radon concentrations were greater than or equal to the EPA’s recommended action level of 4.0 pCi/L of air [4]. We calculated differences in the Lüfts’ and Model 1028 XPs’ 24 h moving averages as the Lüft values subtract the Model 1028 XP values (i.e., positive values meant the Lüft measurements were higher than the Model 1028 XP measurements, whereas negative values meant the Lüft measurements were lower than the Model 1028 XP measurements).

The Lüfts’ 24 h moving averages, Model 1028 XPs 24 h moving averages, and the differences in the Lüfts’ and Model 1028 XPs’ 24 h moving averages were missing for some measurements because the first 23 measurements in each room were not 24 h moving averages, there were suspected brief power outages that affected each room on some days, some measurements were missed in some rooms while we manually downloaded data from the instruments, we had only four Model 1028 XPs when we started the study, there was instrument interference in one room, or other reasons (Supplementary Materials, p. 3). Therefore, we accounted for potential missing data bias using stabilized inverse probability weights (IPWs; Supplementary Materials, p. 3) [19,20,21,22,23,24,25]. We used the IPWs to calculate the frequencies of missing and non-missing values, arithmetic means (AM), standard deviations (SD), minimums, first quartiles, medians, third quartiles, and maximums for the Lüft and Model 1028 XP 24 h moving averages and the differences in the Lüfts’ and Model 1028 XPs’ 24 h moving averages for each of the six air pollutants and environmental measures.

We had between 4845 and 4847 repeated or longitudinal measurements of the Lüfts’ 24 h moving averages, Model 1028 XPs 24 h moving averages, and the differences in the Lüfts’ and Model 1028 XPs’ 24 h moving averages in each of the six rooms in which we collected data for this study (i.e., the repeated measurements were clustered or nested within room [26]). Therefore, we used linear mixed regression models to account for the correlation between the repeated measurements within each room [27,28]. We estimated AM differences in the Lüfts’ and Model 1028 XPs’ 24 h moving averages, 95% confidence intervals (CI), and *p*-values via separate stabilized inverse probability-weighted intercept-only linear mixed regression models that used the differences in the Lüfts’ and Model 1028 XPs’ 24 h moving averages as the response variables, rooms (classroom A, classroom B, custodian’s office, information technology office, library, and music room) as the subjects about which we made repeated measurements, a first order autoregressive moving average (ARMA[1,1]) or first order autoregressive (AR[1]) correlation structure, and robust variance estimates. We estimated the AM differences for barometric pressure, radon, relative humidity, temperature, and VOC because the distributions of the differences of those air pollutants and environmental measures were approximately normal. The distribution of the differences of CO_2_ was right skewed. However, the more appropriate geometric mean (GM) is not defined for negative values [29] and the differences in the Lüfts’ and Model 1028 XPs’ 24 h moving average CO_2_ concentrations had a lot of negative values. Thus, we estimated the AM differences for CO_2_. We used the ARMA(1,1) correlation structure for differences in barometric pressure, CO_2_, relative humidity, temperature, and VOC, and we used the AR(1) correlation structure for differences in radon because those correlation structures had the lowest Akaike Information Criterion (AIC) [30] for differences in those air pollutants and environmental measures of the several correlation structures that we evaluated. We evaluated whether or not to include random intercepts in the stabilized inverse probability-weighted intercept-only linear mixed regression models, but the Wald z-tests of the random intercepts always had *p*-values great than α = 0.05. Thus, we did not include random intercepts in the stabilized inverse probability-weighted intercept-only linear mixed regression models. We calculated Pearson’s correlation coefficients (PCC) and concordance correlation coefficients (CCC) [31] for associations between the Lüft and Model 1028 XP 24 h moving averages for each of the six air pollutants and environmental measures.

We had between 4845 and 4847 repeated or longitudinal measurements of the dichotomous variables for both the Lüfts’ and Model 1028 XPs’ radon concentrations to indicate whether or not the 24 h moving average radon concentrations were greater than or equal to the EPA’s recommended action level of 4.0 pCi/L of air [4] in each of the six rooms in which we collected data for this study. We cross-tabulated the dichotomous variables and calculated the frequencies of measurements in each of the cells of the table. Using the Model 1028 XP radon classification as the gold standard, we modified a SAS macro developed by Ying et al. [32] to incorporate use of IPWs and robust variance estimates and then used the modified SAS macro to calculate stabilized inverse probability-weighted sensitivity, specificity, and 95% CIs for the Lüft radon classification. The modified SAS macro estimated the sensitivity, specificity, and 95% CIs via a stabilized inverse probability-weighted, simple (i.e., unadjusted) unconditional logistic regression model with generalized estimating equations (GEE) that used the Lüft classification as the response variable, the Model 1028 XP classification as the explanatory variable, rooms (classroom A, classroom B, custodian’s office, information technology office, library, and music room) as the subjects about which we made repeated measurements, an independent correlation structure, and robust variance estimates. The modified SAS macro used GEE to account for the correlation between the repeated measurements within each room [26,32,33].

We made line graphs of mean (between rooms) stabilized inverse probability-weighted Lüft and Model 1028 XP 24 h moving averages by time for each of the six air pollutants and environmental measures.

## 3. Results

The AM IPW was 0.96, the minimum was 0.87, and the maximum was 5.42 (Supplementary Materials, Table S1). The AM (SD) stabilized inverse probability-weighted 24 h moving average barometric pressures measured by the Lüfts and Model 1028 XPs were 24.80 (0.17) inHg and 24.78 (0.16) inHg, respectively, and the AM (SD) difference was 0.02 (0.08) inHg (Table 2). The median (interquartile range [IQR]) stabilized inverse probability-weighted 24 h moving average CO_2_ concentrations measured by the Lüfts and Model 1028 XPs were 500.00 (109.00) ppm and 745.88 (484.45) ppm, respectively, and the median (IQR) difference was −222.79 (453.92) ppm. The median (IQR) stabilized inverse probability-weighted 24 h moving average radon concentrations measured by the Lüfts and Model 1028 XPs were 2.02 (1.86) pCi/L of air and 1.98 (1.92) pCi/L of air, respectively, and the AM (SD) difference was −0.02 (0.77) pCi/L of air. The AM (SD) stabilized inverse probability-weighted 24 h moving average relative humidities measured by the Lüfts and Model 1028 XPs were 22.84 (5.07)% and 28.33 (5.47)%, respectively, and the AM (SD) difference was −5.48 (2.31)%. The AM (SD) stabilized inverse probability-weighted 24 h moving average temperatures measured by the Lüfts and Model 1028 XPs were 23.76 (1.72) °C and 21.44 (1.91) °C, respectively, and the AM (SD) difference was 2.32 (0.95) °C. The median (IQR) stabilized inverse probability-weighted 24 h moving average VOC concentrations measured by the Lüfts and Model 1028 XPs were 72.00 (60.00) ppb and 52.58 (81.25) ppb, respectively, and the AM (SD) difference was −46.64 (339.57) ppb.

The AM stabilized inverse probability-weighted differences in the Model 1028 XPs’ and Lüfts’ 24 h moving averages were significantly different from zero for barometric pressure (*p* = 0.02), CO_2_ (*p* = 0.002), relative humidity (*p* < 0.0001), temperature (*p* = 0.0001), and VOC (*p* = 0.007), but not for radon (*p* = 0.50) (Table 3). The CCC for associations between the stabilized inverse probability-weighted Model 1028 XP and Lüft 24 h moving averages for barometric pressure, CO_2_, radon, relative humidity, temperature, and VOC were 0.87, 0.05, 0.92, 0.59, 0.48, and 0.03, respectively. Using the Model 1028 XPs’ classifications of whether or not the 24 h moving average radon concentrations were greater than or equal to the EPA’s recommended action level of 4.0 pCi/L of air [4] as the gold standard, the stabilized inverse probability-weighted sensitivity of the Lüfts’ classifications was 0.81 (95% CI: 0.74, 0.87) and the specificity was 0.96 (95% CI: 0.93, 0.98) (Table 4).

**Figure 1:**
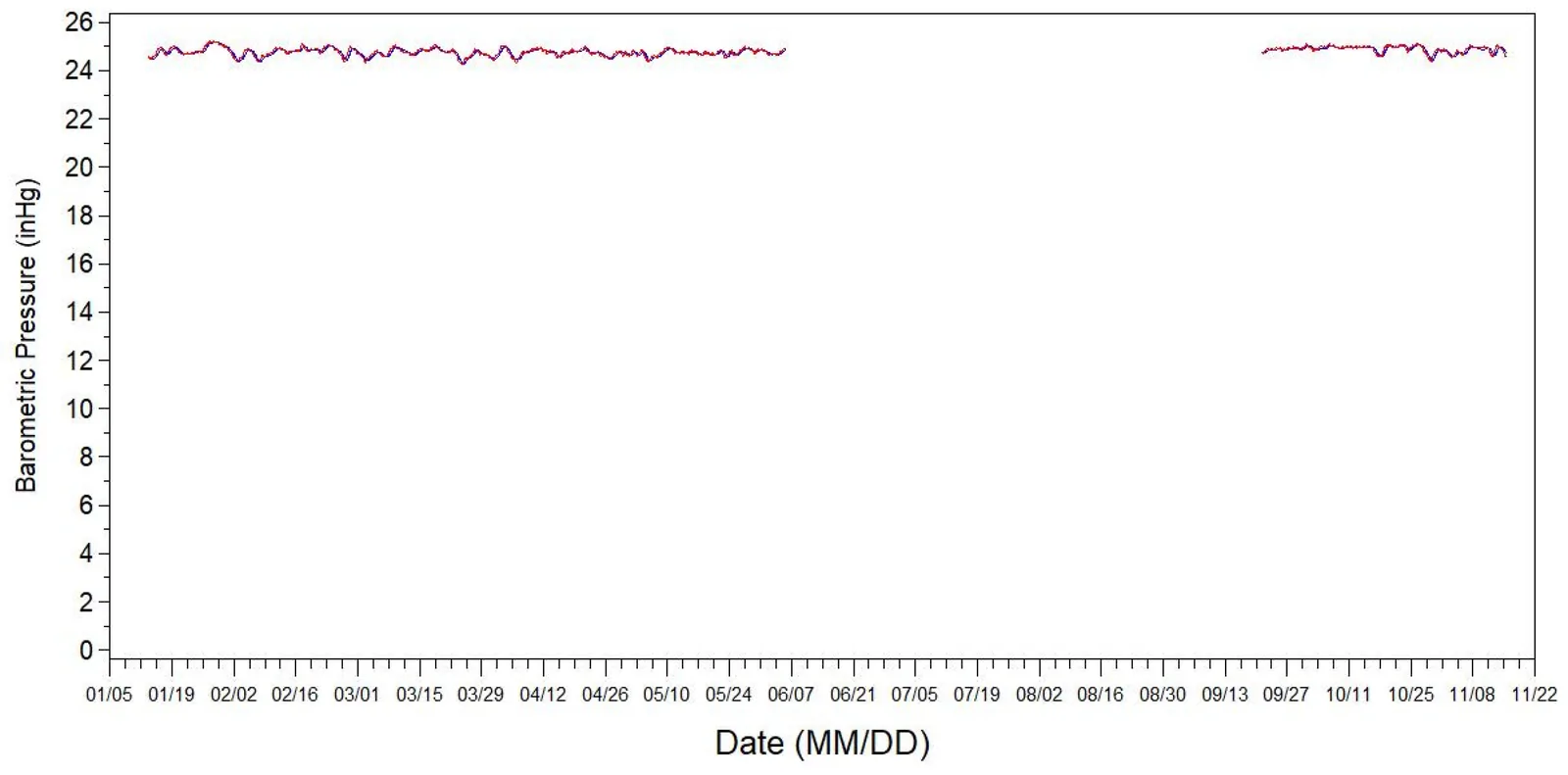
Line graphs of mean (between rooms) stabilized inverse probability-weighted 24 h moving average barometric pressure measured using Lüft (*Sun*RADON LLC, Melbourne, FL, USA) and Model 1028 XP (*Sun*RADON LLC, Melbourne, FL, USA) instruments at a school in Utah, 2024. The red line shows the mean values from the Lüft instruments, whereas the blue line shows the mean values from the Model 1028 XP instruments.

**Figure 2:**
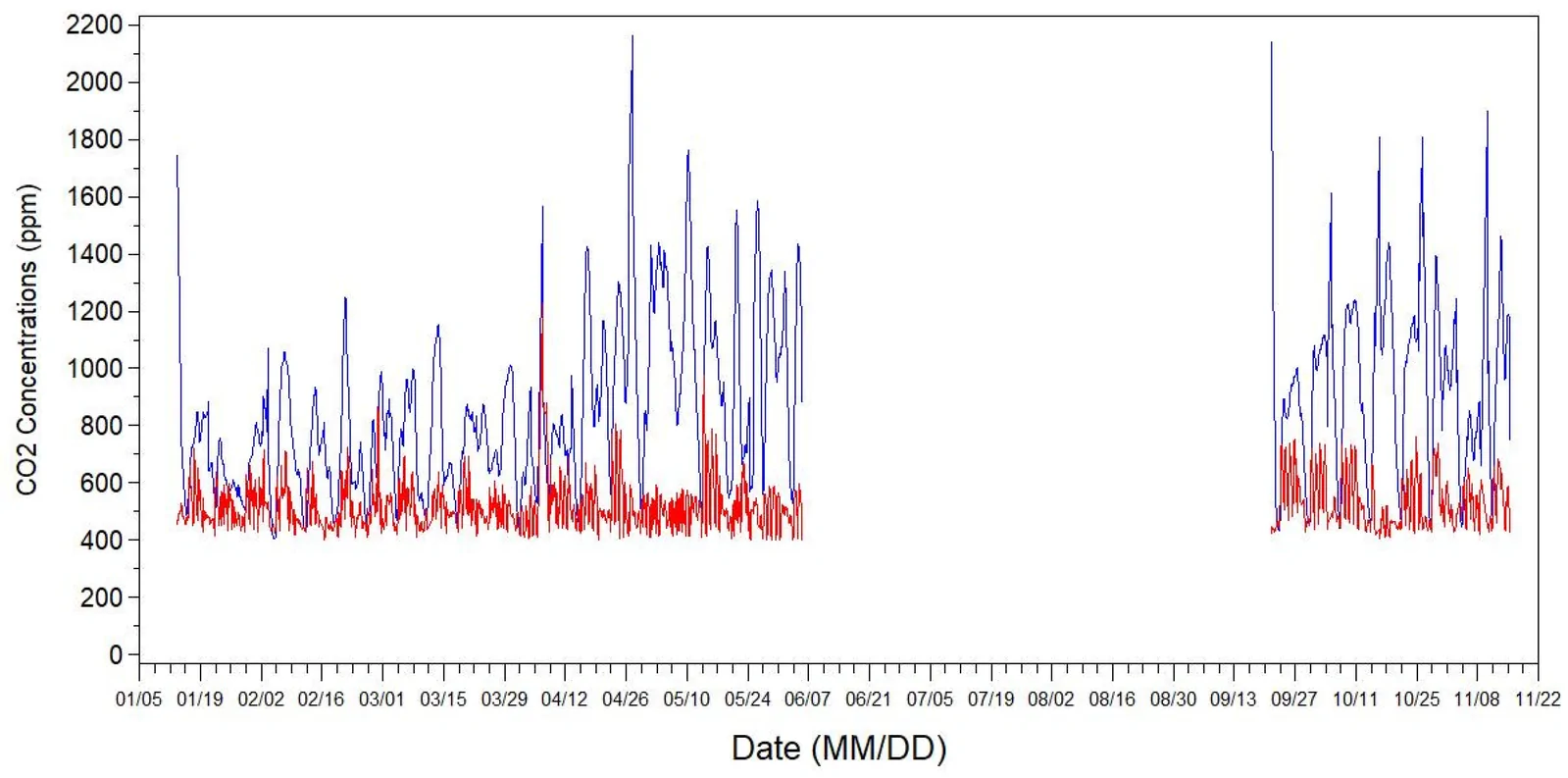
Line graphs of mean (between rooms) stabilized inverse probability-weighted 24 h moving average CO_2_ concentrations measured using Lüft (*Sun*RADON LLC, Melbourne, FL, USA) and Model 1028 XP (*Sun*RADON LLC, Melbourne, FL, USA) instruments at a school in Utah, 2024. The red line shows the mean values from the Lüft instruments, whereas the blue line shows the mean values from the Model 1028 XP instruments. Abbreviations: CO_2_, carbon dioxide.

**Figure 3:**
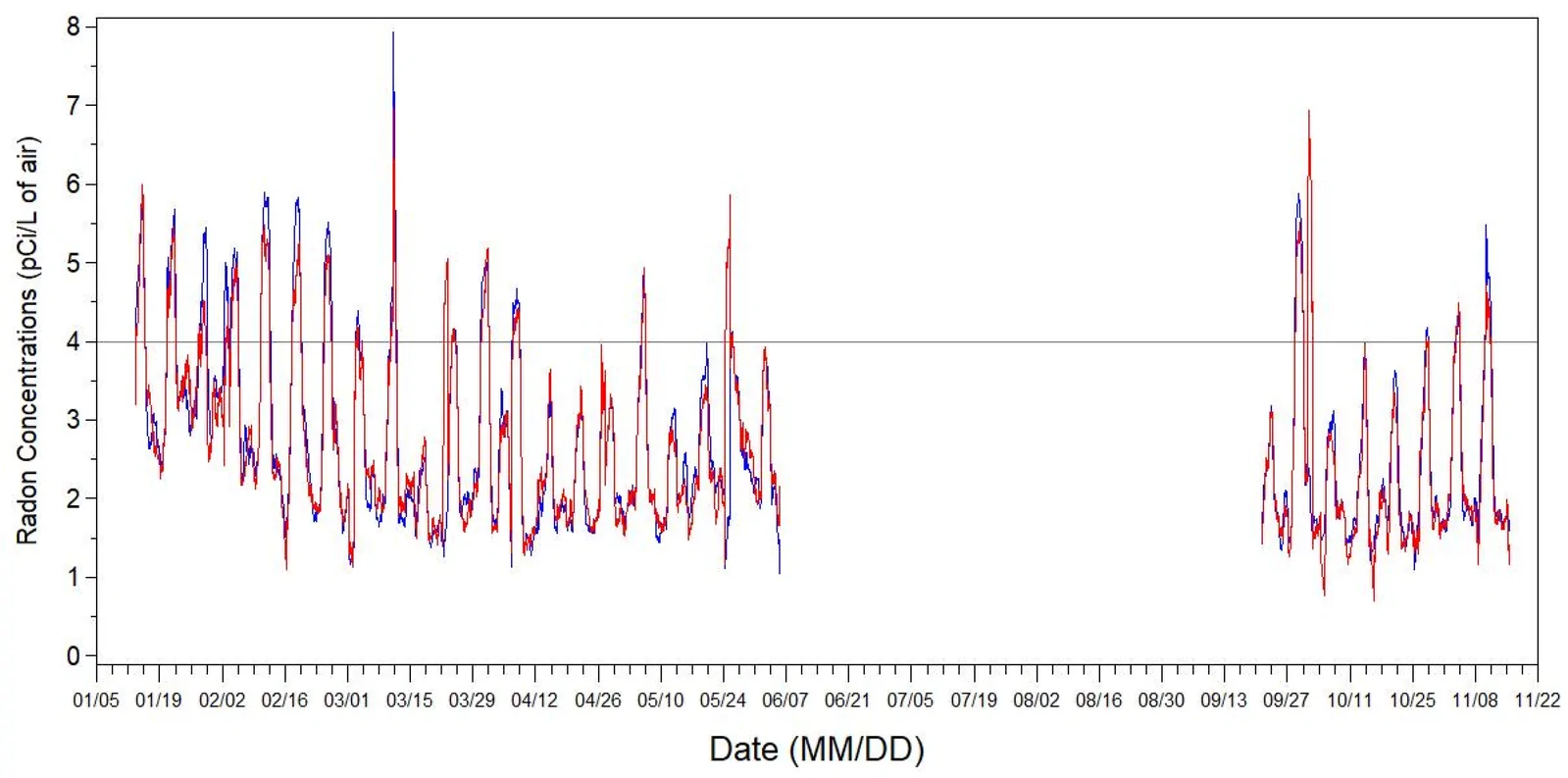
Line graphs of mean (between rooms) stabilized inverse probability-weighted 24 h moving average radon concentrations measured using Lüft (*Sun*RADON LLC, Melbourne, FL, USA) and Model 1028 XP (*Sun*RADON LLC, Melbourne, FL, USA) instruments at a school in Utah, 2024. The red line shows the mean values from the Lüft instruments, whereas the blue line shows the mean values from the Model 1028 XP instruments, and the black line represents the EPA’s recommended action level of 4.0 pCi/L of air [4]. Abbreviations: EPA, U.S. Environmental Protection Agency.

**Figure 4:**
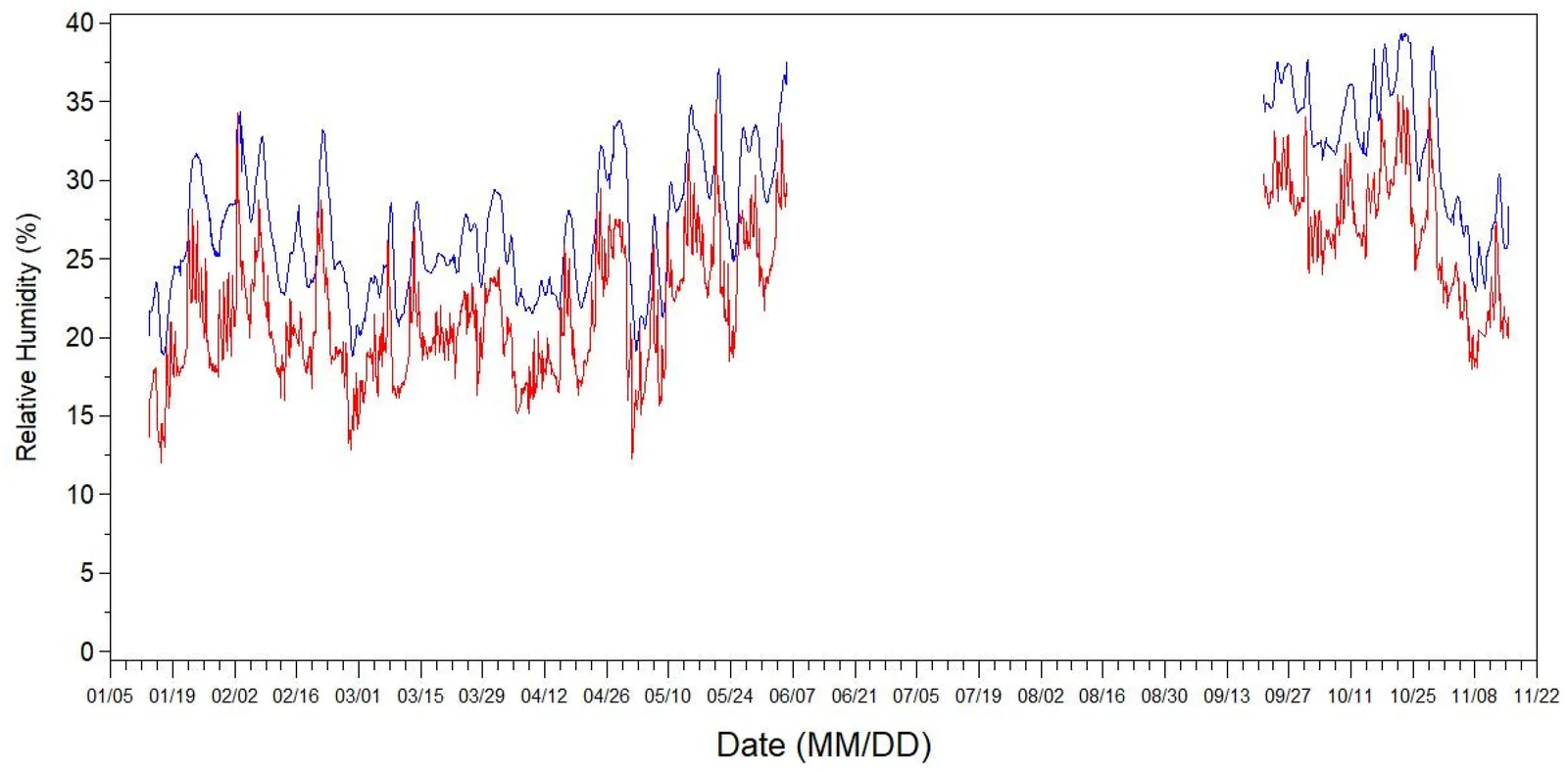
Line graphs of mean (between rooms) stabilized inverse probability-weighted 24 h moving average relative humidity measured using Lüft (*Sun*RADON LLC, Melbourne, FL, USA) and Model 1028 XP (*Sun*RADON LLC, Melbourne, FL, USA) instruments at a school in Utah, 2024. The red line shows the mean values from the Lüft instruments, whereas the blue line shows the mean values from the Model 1028 XP instruments.

**Figure 5:**
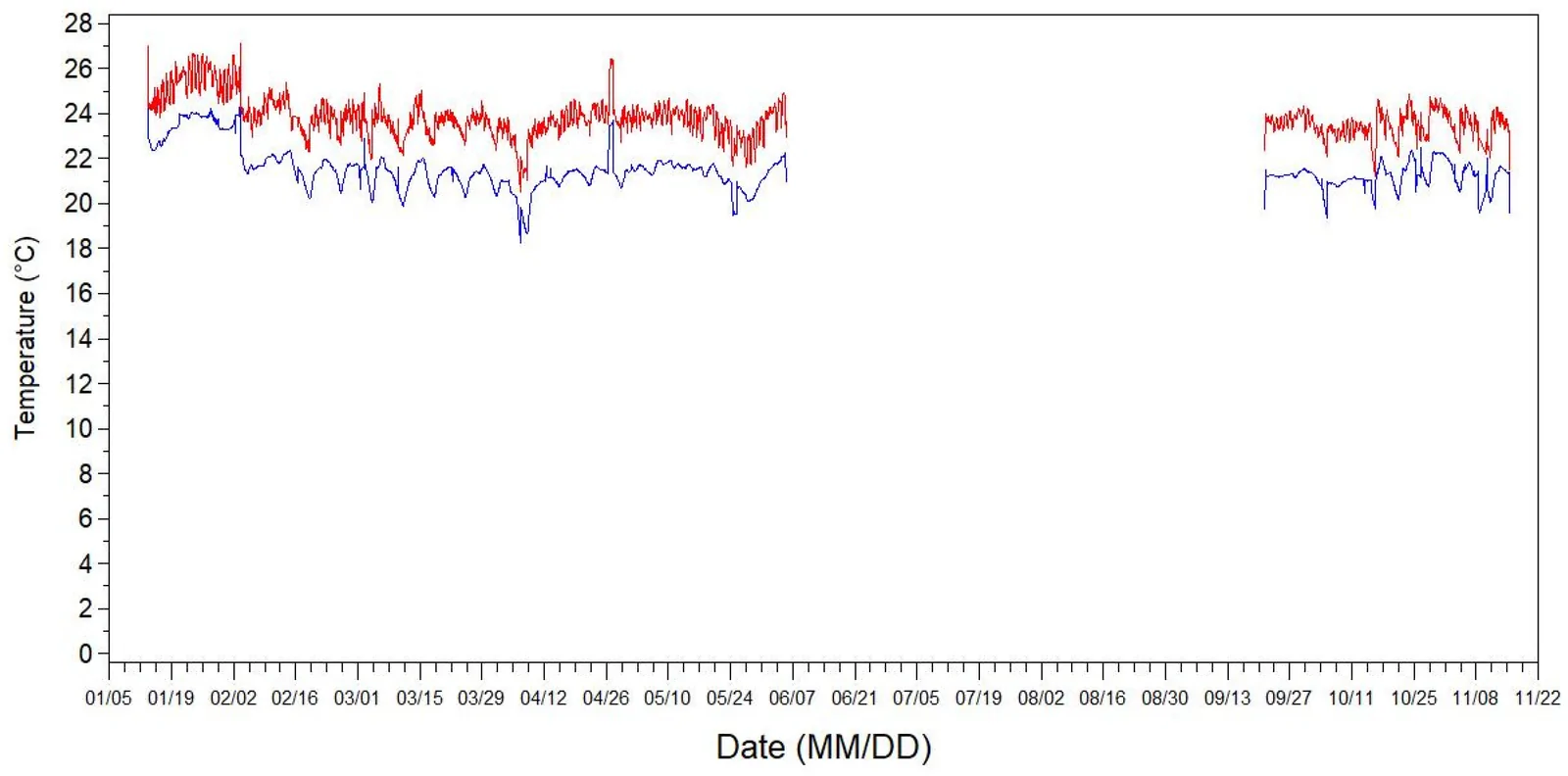
Line graphs of mean (between rooms) stabilized inverse probability-weighted 24 h moving average temperature measured using Lüft (*Sun*RADON LLC, Melbourne, FL, USA) and Model 1028 XP (*Sun*RADON LLC, Melbourne, FL, USA) instruments at a school in Utah, 2024. The red line shows the mean values from the Lüft instruments, whereas the blue line shows the mean values from the Model 1028 XP instruments.

**Figure 6:**
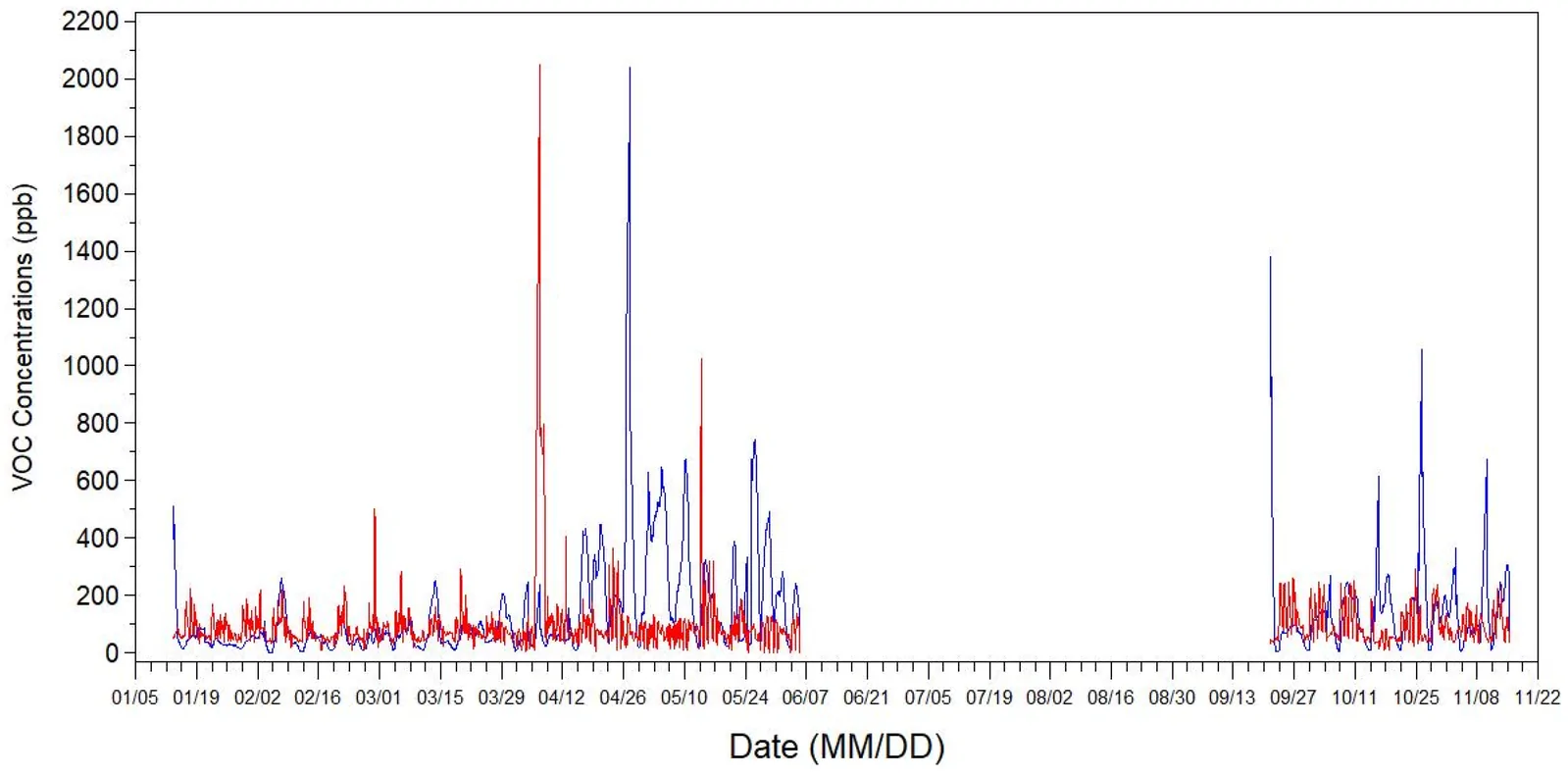
Line graphs of mean (between rooms) stabilized inverse probability-weighted 24 h moving average VOC concentrations measured using Lüft (*Sun*RADON LLC, Melbourne, FL, USA) and Model 1028 XP (*Sun*RADON LLC, Melbourne, FL, USA) instruments at a school in Utah, 2024. The red line shows the mean values from the Lüft instruments, whereas the blue line shows the mean values from the Model 1028 XP instruments. Abbreviations: VOC, volatile organic compounds.

The line graphs of mean (between rooms) stabilized inverse probability-weighted Lüft and Model 1028 XP 24 h moving averages by time indicated short-term changes that generally seemed to correspond with weekdays (Monday–Friday) and weekends (Saturday–Sunday; Figure 1, Figure 2, Figure 3, Figure 4, Figure 5 and Figure 6). There was no discernible, consistent pattern in short-term changes for barometric pressure (Figure 1). Compared to weekdays, barometric pressure increased during some weekends, decreased during other weekends, and had no discernible change during other weekends. Compared to weekdays, radon concentrations (Figure 3) and relative humidity (Figure 4) generally increased during weekends, but CO_2_ concentrations (Figure 2), temperature (Figure 5), and VOC concentrations (Figure 6) generally decreased during weekends.

The line graphs of mean (between rooms) stabilized inverse probability-weighted Lüft and Model 1028 XP 24 h moving averages by time indicated different long-term patterns for each of the six air pollutants and environmental measures. Barometric pressures were relatively constant over time, and there was little separation in the measurements made by the Lüfts and Model 1028 XPs (Figure 1). CO_2_ concentrations measured by the Model 1028 XPs increased over time, but CO_2_ concentrations measured by the Lüfts were relatively constant over time and were consistently lower than the CO_2_ concentrations measured by the Model 1028 XPs (Figure 2). Radon concentrations decreased until approximately the middle of April, were relatively constant until approximately the end of October, and then increased (Figure 3). There was little separation in the measurements of radon concentrations made by the Lüfts and Model 1028 XPs. Relative humidities were relatively constant until approximately the end of April, were increased until approximately the end of October, and then decreased (Figure 4). Relative humidities measured by the Lüfts were consistently lower than the relative humidities measured by the Model 1028 XPs. Temperatures decreased at approximately the beginning of February and were relatively constant thereafter (Figure 5). Temperatures measured by the Lüfts were consistently higher than the temperatures measured by the Model 1028 XPs. Other than a few isolated increases, VOC concentrations measured by the Lüfts were relatively constant over time (Figure 6). VOC concentrations measured by the Model 1028 XPs were increased from approximately the middle of April until the beginning of June and from approximately the middle of October until the middle of November but were relatively constant otherwise. VOC concentrations measured by the Lüfts were similar to VOC concentrations measured by the Model 1028 XPs except for the aforementioned times when the VOC concentrations measured by the Model 1028 XPs were increased. The VOC concentrations measured by the Lüfts also had fewer isolated increases than the VOC concentrations measured by the Model 1028 XPs.

## 4. Discussion

In this validation study of a consumer- vs. industrial-grade indoor radon and air quality monitor at a school in Utah, we found the consumer-grade monitor—the Lüft—provided 24 h moving averages of barometric pressure and radon concentrations that were very similar to the 24 h moving averages provided by the industrial-grade monitor—the Model 1028 XP. The Lüfts’ classifications of whether or not the 24 h moving average radon concentrations were greater than or equal to the EPA’s recommended action level of 4.0 pCi/L of air [4] were similar to the Model 1028 XPs’ classifications. However, the 24 h moving averages of CO_2_ concentrations, relative humidities, and VOC concentrations provided by the Lüft were usually lower than the 24 h moving averages provided by the Model 1028 XP, although this was less true for VOC concentrations. The 24 h moving averages of temperatures provided by the Lüft were usually higher than the 24 h moving averages provided by the Model 1028 XP.

The American Society of Heating, Refrigerating and Air-Conditioning Engineers (ASHRAE) recommends indoor CO_2_ concentrations be kept below 700 ppm above outdoor CO_2_ concentrations [34] (i.e., approximately 1000 ppm [7]). The median 24 h moving average CO_2_ concentration measured by the Model 1028 XPs in our study was 745.88 ppm, but 28% of the Model 1028 XPs’ 24 h moving average CO_2_ concentrations were greater than or equal to 1000 ppm. Fifteen percent of the Model 1028 XPs’ 24 h moving average radon concentrations were greater than or equal to the EPA’s recommended action level of 4.0 pCi/L of air [4], although the median 24 h moving average radon concentration measured by the Model 1028 XPs in our study was 1.98 pCi/L of air. The AM 24 h moving average relative humidity measured by the Model 1028 XPs in our study was 28.33%, and 59% of the Model 1028 XPs’ 24 h moving average relative humidities were below EPA’s recommendation of 30–60% [7]. ASHRAE’s recommendations for indoor temperature vary based on time of year and relative humidity [35], but the minimum, 15.98 °C, and maximum, 27.73 °C, 24 h moving average temperatures measured by the Model 1028 XP’s in our study were below and above, respectively, the most extreme minimum, 67.5 °F (19.72 °C), and maximum, 80 °F (26.67 °C), temperatures recommended by ASHRAE. EPA has not provided a recommended concentration below which indoor VOC concentrations should be kept [36]. Regardless, the non-negligible proportions of 24 h moving averages of the six air pollutants and environmental measures included in our study that were outside of EPA’s and ASHRAE’s recommendations suggest improvements are needed in the indoor air quality of the school in which we conducted this study.

No study has assessed the validity of the Lüft’s measurements of barometric pressure, CO_2_ concentrations, relative humidity, temperature, or VOC concentrations. Only two previous studies assessed the validity of the Lüft’s measurements of radon concentrations in comparison to an industrial-grade reference monitor [12,13], although neither study assessed the validity of the Lüft’s classifications of whether or not radon concentrations were greater than or equal to some recommended value, such as the EPA’s recommended action level of 4.0 pCi/L of air [4]. Lemieux et al. [12] conducted two separate assessments of the validity of the Lüft’s measurements of radon concentrations. Results from six Lüfts were averaged for each assessment. The first assessment compared the Lüfts’ measurements of radon concentrations at 200 becquerels per cubic meter of air (Bq/m^3^) (5.41 pCi/L) and 1400 Bq/m^3^ (37.84 pCi/L) to the AlphaGUARD PQ2000’s (Bertin GmbH, Frankfurt, Germany) measurements in a 0.5 m^3^ sealed radon chamber in a laboratory in Ottawa, ON, Canada. Two weeks of data were collected hourly at both radon concentrations. At 200 Bq/m^3^, the Lüfts’ measurements of radon concentrations were an average of approximately 30 Bq/m^3^ (0.81 pCi/L) below the AlphaGUARD PQ2000’s measurements. At 1400 Bq/m^3^, the Lüfts’ measurements of radon concentrations were an average of approximately 108 Bq/m^3^ (2.92 pCi/L) below the AlphaGUARD PQ2000’s measurements. The second assessment compared the Lüfts’ measurements of radon concentrations at an average concentration of 2443 Bq/m^3^ (66.03 pCi/L) to the AlphaGUARD DF2000’s (Bertin GmbH, Frankfurt, Germany) measurements in a 12 m^3^ walk-in radon chamber in a laboratory in Saskatoon, SK, Canada. Over three weeks of hourly data collection, the Lüfts’ measurements of radon concentrations were an average of approximately 58 Bq/m^3^ (1.56 pCi/L) below the AlphaGUARD DF2000’s measurements. Bahadori and Hanson [13] evaluated the performance of the Lüft at three radon concentrations, 0.63, 15.5, and 29.4 pCi/L of air, using averaged results from five Lüfts after seven days of hourly data collection at each radon concentration. The lowest radon concentration was evaluated in a room in Manhattan, KS, USA. Compared to the Radon Eye Pro’s (Ecosense, San Jose, CA, USA) measurements, the Lüfts’ measurements of radon concentrations were an average of approximately 0.07 pCi/L of air lower. The middle and highest radon concentrations were evaluated in a 0.46 m^3^ sealed radon chamber in a laboratory in Manhattan, KS, USA. Compared to the Pylon AB-5′s (Pylon Electronics Inc., Ottawa, ON, Canada) measurements, the Lüfts’ measurements of radon concentrations were an average of approximately 3.16 pCi/L of air lower for the middle concentration and 3.72 pCi/L of air lower for the highest concentration. In our study, the Lüfts’ measurements of 24 h moving average radon concentrations were an average of approximately 0.05 pCi/L of air below the Model 1028 XPs’ measurements over approximately 29 weeks of data collection in each of six rooms at a school in Utah that experienced radon concentrations that ranged from 0.44 to 22.49 pCi/L of air according to the Model 1028 XPs. The differences in results between the studies conducted by Lemieux et al. [12], Bahadori and Hanson [13], and our study may be explained by differences in industrial-grade reference instruments, lengths and locations of data collection, and ranges of radon concentrations utilized by the three studies.

Definitive reasons for discrepancies between the Lüfts’ and Model 1028 XPs’ 24 h moving averages of CO_2_, relative humidity, temperature, and VOC are not immediately apparent. The Lüfts detected the same VOCs as the Model 1028 XPs [17] and used the same hardware and approach (i.e., self-baselining) that had the same measurement range for measuring VOCs and CO_2_ as the Model 1028 XPs. However, self-baselining occurs at various times and can lead to unexpected increases or decreases in the reported relative CO_2_ and VOC concentrations measured by the Lüfts and Model 1028 XPs. If self-baseline and the resultant unexpected increases or decreases in the reported relative CO_2_ and VOC concentrations measured by the Lüfts and Model 1028 XPs occurred at different times for the Lüfts and Model 1028 XPs, then self-baselining might explain at least some of the discrepancies between the Lüfts’ and Model 1028 XPs’ 24 h moving averages of CO_2_ and VOC. The Lüfts used a different hardware and approach with different measurement ranges for measuring relative humidity and temperature than the Model 1028 XPs, which may help explain the discrepancies between the Lüfts’ and Model 1028 XPs’ 24 h moving averages of relative humidity and temperature. Considering the known, typically inverse association between indoor relative humidity and indoor temperature [37], it is not surprising that the Lüfts’ measurements were high compared to the Model 1028 XPs’ measurements for one of these environmental measurements (temperature in this case) and low for the other (relative humidity in this case).

The short-term patterns in 24 h moving averages of each of the six air pollutants and environmental measures included in our study were consistent with those observed in other studies [15,38,39]. Increases in CO_2_ concentrations during weekdays were likely primarily due to exhalation of people in the school and secondarily due to CO_2_ from vehicular traffic being brought into the school by the HVAC system, whereas decreases in people in the school and vehicular traffic near the school were the likely reasons CO_2_ concentrations decreased during weekends [6,7]. Radon concentrations were decreased during weekdays likely because the school’s HVAC system replaced inside air with outside air when it was on. Radon concentrations were increased during the weekends, likely because radon entered the school via cracks in the foundation or walls, holes in the floor, or drains [2], and there was minimal or no air exchange between inside and outside of the school because the HVAC system was off. Although we could not detect cracks in the foundation or walls because most floors were covered with carpet or tile, we did observe holes in the floor of the information technology office that allowed wires and cables into the room, and a drain in the custodian’s office—two of the rooms in which we collected data for this study. Increases in VOC concentrations during weekdays were likely due to the use of adhesives, markers, paints, or cleaners by people in the school, whereas decreases in people in the school was a likely reason VOC concentrations decreased during weekends [7]. The long-term pattern we observed in the 24 h moving average radon concentrations is consistent with previous studies that found seasonal changes in indoor radon concentrations, including increased radon concentrations when the outdoor temperature decreased and decreased radon concentrations when the outdoor temperature increased [40].

Our findings suggest the Lüft is a valid monitor for measuring 24 h moving average indoor radon concentrations, barometric pressure, and whether or not the 24 h moving average radon concentrations are greater than or equal to the EPA’s recommended action level of 4.0 pCi/L of air [4]. The Lüft is also easy to use, more affordable, and user-friendly and it does not require calibration annually by the manufacturer. Therefore, the Lüft is a good option for monitoring long-term radon concentrations in schools. However, the Lüft should not be used if measurements of radon concentrations averaged for less than 24 h are desired, such as the time when people are inside schools. In addition, our findings suggest the Lüft is not a valid monitor for measuring 24 h moving average indoor CO_2_ concentrations, relative humidity, temperature, and VOC concentrations. Therefore, the Lüft should not be used if measurements of long-term indoor CO_2_, relative humidity, temperature, and/or VOC are of interest.

Our study had several strengths including confounding by time-fixed factors (e.g., differences between rooms) was controlled by design because the Lüft and Model 1028 XP were co-located within a few inches of each other in each of the six rooms of the school in which we conducted the study. We could evaluate the long-term accuracy of the Lüft because we collected data for approximately 29 weeks during four seasons. Our results likely generalize to a wide range of radon concentrations because one of the rooms in which we collected data had an initial radon test result less than the EPA’s recommended action level of 4.0 pCi/L of air [4], and five rooms had initial radon test results greater than or equal to 4.0 pCi/L of air. Furthermore, the 24 h moving average radon concentrations measured by the Model 1028 XPs ranged from 0.44 to 22.49 pCi/L of air. The Model 1028 XP is calibrated by the manufacturer annually and is industrial-grade, so it was a good choice for a gold standard. Our results for radon concentrations were robust to the outcome definition because we obtained similar results when we analyzed the 24 h moving averages of radon concentrations and whether or not the 24 h moving average radon concentrations were greater than or equal to the EPA’s recommended action level of 4.0 pCi/L of air [4]. We were able to account for correlations between measurements made in the same rooms by using linear mixed regression models.

Our study also had some limitations, including that our results may not generalize to other schools or other types of buildings, because we collected data from only one school. We could not estimate the GM differences in the Lüfts’ and Model 1028 XPs’ 24 h moving average CO_2_ concentrations and had to estimate the AM instead. It would have been more appropriate to calculate the repeated measures CCC than the CCC because the repeated measures CCC could have appropriately accounted for within-room correlations from the repeated measures [41,42]. In addition, a SAS macro, %rm_CCC, that can be used to calculate the repeated measures CCC has been published [43]. However, we did not have enough computer memory to successfully calculate the repeated measures CCC using the published SAS macro. Similarly, we could not use published SAS macros [32] to provide estimates of the positive predictive value and negative predictive value of the Lüfts’ classifications of whether or not the 24 h moving average radon concentrations were greater than or equal to the EPA’s recommended action level of 4.0 pCi/L of air [4] because we did not have enough computer memory. We could not compare averages of the six air pollutants and environmental measures measured by the Lüfts and Model 1028 XPs for time periods less than 24 h because the Lüfts provided only 24 h moving averages. Furthermore, we could not calculate the hourly averages from the Lüfts’ 24 h moving averages because there were two unknowns in the equation needed to complete the calculation. The differences in the Lüfts’ and Model 1028 XPs’ 24 h moving averages were missing for 3885 (13%) of measurements, so we used IPWs to account for potential missing data bias [19,20,21,22]. There were no IPWs less than 0.05 or greater than 20.00, but the AM IPW could have been closer to 1.00 and we were unable to improve the AM IPW using alternative unconditional logistic regression models to calculate the IPWs.

## 5. Conclusions

In conclusion, using the Model 1028 XP as the gold standard, the Lüft made accurate measurements of 24 h moving averages of barometric pressures, 24 h moving averages of radon concentrations, and whether or not the 24 h moving average radon concentrations were greater than or equal to the EPA’s recommended action level of 4.0 pCi/L of air [4]. However, the Lüft may not have provided accurate measurements of 24 h moving averages of CO_2_ concentrations, relative humidity, temperature, and VOC concentrations. In addition, the Lüft did not give hourly averages of the six air pollutants and environmental measures. Therefore, the Lüft should not be used if averages for time periods less than 24 h are desired, which seems particularly important for schools, workplaces, or businesses because the interest may be in whether concentrations of radon and/or other indoor air quality parameters are low and/or acceptable during times when people are in the buildings. However, considering the cost of the Lüft was approximately one-fifth to one-fourth of the cost of the Model 1028 XP when we purchased the monitors and the Lüft does not require annual calibration by the manufacturer like the Model 1028 XP does, the Lüft should likely be a desirable option for monitoring radon concentrations in buildings over long time periods. Future research should replicate this study in other schools and other types of buildings including homes.

## Figures and Tables

**Table 1:** Measurement ranges for radon and indoor air quality parameters that can be measured by the Lüft (*Sun*RADON LLC, Melbourne, FL, USA) and Model 1028 XP (*Sun*RADON LLC, Melbourne, FL, USA) instruments [16,17,18].

	Measurement Ranges	
Variable	Lüft	Model 1028 XP
Barometric pressure, inHg	23.62–32.48	23.62–32.48
CO_2_, ppm	400–8192	400–8192
Radon, pCi/L of air	0–9999	0.1–9999
Relative humidity, %	10–95	10–90
Temperature, °C	4.44–40.56	7.22–35.00
VOC, ppb	0–1187	0–1187

Abbreviations: CO_2_, carbon dioxide; VOC, volatile organic compounds.

**Table 2:** Summary statistics for stabilized inverse probability-weighted 24 h moving average data measured using Lüft (*Sun*RADON LLC, Melbourne, FL, USA) and Model 1028 XP (*Sun*RADON LLC, Melbourne, FL, USA) instruments at a school in Utah, 2024.

Variable	Missing, n	Not Missing, n	AM	SD	Min	Q1	Median	Q3	Max
Lüft barometric pressure, inHg	1088	27,986	24.80	0.17	24.26	24.69	24.80	24.92	25.24
Model 1028 XP barometric pressure, inHg	3095	25,979	24.78	0.16	24.27	24.68	24.78	24.90	25.18
Barometric pressure difference, inHg	3885	25,189	0.02	0.08	−0.23	−0.04	0.01	0.07	0.34
Lüft CO_2_, ppm	1088	27,986	521.61	93.76	400.00	454.00	500.00	563.00	1290.00
Model 1028 XP CO_2_, ppm	3095	25,979	869.18	420.52	400.58	566.88	745.88	1051.33	3617.42
CO_2_ difference, ppm	3885	25,189	−347.57	412.35	−3088.42	−510.50	−222.79	−56.58	457.92
Lüft radon, pCi/L of air	1088	27,986	2.61	1.86	0.32	1.37	2.02	3.23	20.10
Model 1028 XP radon, pCi/L of air	3095	25,979	2.63	1.96	0.44	1.35	1.98	3.27	22.49
Radon difference, pCi/L of air	3885	25,189	−0.02	0.77	−3.74	−0.34	−0.02	0.26	9.78
Lüft relative humidity, %	1088	27,986	22.84	5.07	9.40	18.92	22.21	26.59	40.54
Model 1028 XP relative humidity, %	3095	25,979	28.33	5.47	14.04	24.13	27.75	32.46	44.33
Relative humidity difference, %	3885	25,189	−5.48	2.31	−16.84	−6.95	−5.53	−4.17	8.47
Lüft temperature, °C	1088	27,986	23.76	1.72	18.51	22.66	23.42	24.59	30.66
Model 1028 XP temperature, °C	3095	25,979	21.44	1.91	15.98	19.95	20.97	23.03	27.73
Temperature difference, °C	3885	25,189	2.32	0.95	−2.85	1.73	2.42	2.93	8.76
Lüft VOC, ppb	1088	27,986	91.81	98.09	0.00	48.00	72.00	108.00	2392.00
Model 1028 XP VOC, ppb	3095	25,979	138.45	331.18	0.00	24.67	52.58	105.92	4175.75
VOC difference, ppb	3885	25,189	−46.64	339.57	−4087.75	−29.04	24.21	53.75	2298.79

Abbreviations: AM, arithmetic mean; CO_2_, carbon dioxide; Q1, first quartile; Max, maximum; Min, minimum; SD, standard deviation; Q3, third quartile; VOC, volatile organic compounds.

**Table 3:** Differences in stabilized inverse probability-weighted 24 h moving average data measured using Lüft (*Sun*RADON LLC, Melbourne, FL, USA) and Model 1028 XP (*Sun*RADON LLC, Melbourne, FL, USA) instruments at a school in Utah, 2024.

Variable	AM ^a^	95% CI ^a^	*p*-Value ^a^	PCC	CCC
Barometric pressure difference, inHg	0.03	0.01, 0.06	0.02	0.88	0.87
CO_2_ difference, ppm	−410.53	−597.69, −223.37	0.002	0.20	0.05
Radon difference, pCi/L of air	−0.05 ^b^	−0.24, 0.14 ^b^	0.50 ^b^	0.92	0.92
Relative humidity difference, %	−5.87	−7.05, −4.69	<0.0001	0.91	0.59
Temperature difference, °C	2.32	1.77, 2.87	0.0001	0.87	0.48
VOC difference, ppb	−194.74	−308.82, −80.66	0.007	0.06	0.03

Abbreviations: AM, arithmetic mean; CO_2_, carbon dioxide; CI, confidence interval; CCC, concordance correlation coefficient; AR(1), first order autoregressive; ARMA(1,1), first order autoregressive moving average; PCC, Pearson’s correlation coefficient; VOC, volatile organic compounds. ^a^ Estimated via a stabilized inverse probability-weighted intercept-only linear mixed regression model that used the original values as the outcome, rooms (classroom A, classroom B, custodian’s office, information technology office, library, and music room) as the subjects about which we made repeated measurements, a ARMA(1,1) correlation structure, and robust variance estimates. ^b^ Estimated via a stabilized inverse probability-weighted intercept-only linear mixed regression model that used the original values as the outcome, rooms (classroom A, classroom B, custodian’s office, information technology office, library, and music room) as the subjects about which we made repeated measurements, a AR(1) correlation structure, and robust variance estimates.

**Table 4:** Accuracy of stabilized inverse probability-weighted 24 h moving average radon data measured using the Lüft (*Sun*RADON LLC, Melbourne, FL, USA) instrument compared to the Model 1028 XP (*Sun*RADON LLC, Melbourne, FL, USA) at a school in Utah, 2024.

	Model 1028 XP						
Lüft	Radon ≥ 4.0 pCi/L of Air ^a^						
Radon ≥ 4.0 pCi/L of Air	Yes	No	Total	Se ^b^	95% CI ^b^	Sp ^b^	95% CI ^b^
Yes	3216	815	4031	0.81	0.74, 0.87	0.96	0.93, 0.98
No	730	20,428	21,158				
Total	3946	21,243	25,189				

Abbreviations: CI, confidence interval; Se, sensitivity; Sp, specificity; EPA, U.S. Environmental Protection Agency. ^a^ The EPA’s recommended action level is 4.0 pCi/L of air [4]. ^b^ Estimated via a stabilized inverse probability-weighted, simple (i.e., unadjusted) unconditional logistic regression model with generalized estimating equations that used the Lüft classification as the dependent variable, the Model 1028 XP classification as the independent variable, rooms (classroom A, classroom B, custodian’s office, information technology office, library, and music room) as the subjects about which we made repeated measurements, an independent correlation structure, and robust variance estimates [32].

## Data Availability

The data that support the findings of this study are available from the corresponding author upon reasonable request.

## Supplementary Materials

The following supporting information can be downloaded at: https://www.mdpi.com/article/10.3390/s26165230/s1, Supplementary Methods; Supplementary Statistical Analyses; Table S1. Summary statistics for stabilized inverse probability weights used to account for potential missing data bias in an analysis of 24 h moving average data measured using Lüft (*Sun*RADON LLC, Melbourne, FL, USA) and Model 1028 XP (*Sun*RADON LLC, Melbourne, FL, USA) instruments at a school in Utah, 2024; References.

## Abbreviations

The following abbreviations are used in this manuscript:

AIC	Akaike Information Criterion
AM	Arithmetic mean
AR(1)	First order autoregressive
ARMA(1,1)	First order autoregressive moving average
ASHRAE	American Society of Heating, Refrigerating and Air-Conditioning Engineers
Bq/m^3^	Becquerels per cubic meter
CCC	Concordance correlation coefficient
CI	Confidence interval
CO_2_	Carbon dioxide
CRM	Continuous radon monitor
EPA	U.S. Environmental Protection Agency
GM	Geometric mean
HVAC	Heating, ventilation, and air conditioning
IPW	Inverse probability weight
IQR	Interquartile range
IT	Information technology
Max	Maximum
Min	Minimum
PCC	Pearson’s correlation coefficient
pCi/L	Picocuries per liter
Q1	First quartile
Q3	Third quartile
^222^Rn	Radon-222
SD	Standard deviation
Se	Sensitivity
Sp	Specificity
USD	U.S. dollar
VOC	Volatile organic compounds
