# Supplementary material for: Long-Term Validation of a Consumer- vs. Industrial-Grade Indoor Radon and Air Quality Monitor at a School in Utah
Source: Sensors (Basel). 2026 Aug 18;26(16):5230. doi: 10.3390/s26165230 (PMC13517794; doi:10.3390/s26165230)
Supplement: Supplementary file 1 [file sensors-26-05230-s001.zip › sensors-4422997-supplementary.pdf]

Supplementary Materials

Article

Long-Term Validation of a Consumer- vs. Industrial-Grade Indoor Radon and Air Quality Monitor at a School in Utah

John D. Beard [1,*], Maren M. Ball [1], Christopher Kitras [2] and Philip B. Lundrigan [2]

[1] Department of Public Health, Brigham Young University, Provo, UT 84602, USA
[2] Department of Electrical and Computer Engineering, Brigham Young University, Provo, UT 84602, USA
* Correspondence: john_beard@byu.edu; Tel.: +1-801-422-7491

Table of Contents

Supplementary Methods 3
Supplementary Statistical Analyses 3
Table S1. Summary statistics for stabilized inverse probability weights used to account for potential missing data bias in an analysis of 24 h moving average data measured using Lüft (*Sun*RADON LLC, Melbourne, FL, USA) and Model 1028 XP (*Sun*RADON LLC, Melbourne, FL, USA) instruments at a school in Utah, 2024........ 4
References 5

Supplementary Methods

Supplementary Statistical Analyses

The Lüfts' (*Sun*RADON LLC, Melbourne, FL, USA) 24-hour moving averages were missing for 1,088 (4%) measurements because the first 23 measurements in each room reported by the Lüft were not 24-hour moving averages, there were suspected brief power outages on February 15, March 11, April 5, October 4, October 15, and November 9 that affected each room, or other reasons (i.e., for sporadic, single, isolated measurements that were missing). The Model 1028 XPs' (*Sun*RADON LLC, Melbourne, FL, USA) 24-hour moving averages were missing for 3,095 (11%) measurements because we could not calculate 24-hour moving averages using the first 23 hourly measurements in each room, some measurements were missed in some rooms while we manually downloaded data from the Model 1028 XPs on February 2, March 1, April 12, April 26, May 24, June 6, October 4, October 25, or November 8, there were no Model 1028 XPs in classroom A and classroom B January 12-February 2 because we had only four Model 1028 XPs when we started the study, the Model 1028 XP in the information technology office did not record data April 4-12 for unknown reasons (we suspect it was unplugged or tampered with, but did not record the reason at the time), there was interference January 20-February 20 from a Model 1028 XP Long-Term Evolution (i.e., cellular) module in the music room until we replaced the Model 1028 XP with one that did not have a Long-Term Evolution module, or other reasons (i.e., for sporadic, single, isolated measurements that were missing). The differences in the Lüfts' and Model 1028 XPs' 24-hour moving averages were missing for 3,885 (13%) of measurements. Therefore, we accounted for potential missing data bias using stabilized inverse probability weights (IPWs) [1-4]. We calculated the IPWs by dividing the marginal probability of missing data by the conditional probabilities of missing data. We used an intercept-only unconditional logistic regression model that had a dichotomous variable (Yes, No) that indicated whether or not the data were missing as the response variable to calculate the marginal probability of missing data. We used a multivariable unconditional logistic regression model that had the same dichotomous missing data indicator variable as the response variable and Lüft barometric pressure (centered [at the mean: 24.80 inHg], restricted quadratic regression spline with five unequally spaced knots based on measurements with missing data only with knots at -0.36, -0.10, -0.01, 0.11, and 0.36 inHg), Lüft relative humidity (centered [at the mean: 22.86 %], restricted quadratic regression spline with six unequally spaced knots based on measurements with missing data only with knots at -8.88, -4.30, -2.50, -0.25, 3.16, and 7.00 %), and Lüft volatile organic compounds (categorical variable based on quintiles [i.e., 0 to 44, 45 to 62, 63 to 84, 85 to 122, and 123 to 4,939 ppb]) as explanatory variables to calculate the conditional probabilities of missing data. We evaluated including different and additional explanatory variables and different versions of the explanatory variables (e.g., linear; linear and quadratic; linear, quadratic, and cubic; categorical; restricted quadratic regression splines [5]) in the multivariable unconditional logistic regression model we used to calculate the conditional probabilities of missing data. We used the explanatory variables and versions of those explanatory variables that produced IPWs with arithmetic mean nearest to 1.00 and no IPW less than 0.05 or greater than 20.00 [1]. We utilized the IPWs for analyses in the same way sampling weights are utilized to analyze data from complex survey sampling study designs [1,6]. To account for the correlation introduced by the fact the IPWs are calculated from the data, we used robust variance estimates [7].

Table S1. Summary statistics for stabilized inverse probability weights used to account for potential missing data bias in an analysis of 24 h moving average data measured using Lüft (*Sun*RADON LLC, Melbourne, FL, USA) and Model 1028 XP (*Sun*RADON LLC, Melbourne, FL, USA) instruments at a school in Utah, 2024.

Variable	c statistic	AM	SD	Min	Q1	Median	Q3	Max
Missing data	0.65	0.96	0.11	0.87	0.93	0.95	0.97	5.42

Abbreviations: AM, arithmetic mean; Q1, first quartile; Max, maximum; Min, minimum; SD, standard deviation; Q3, third quartile.

References

1. Cole, S. R.; Hernan, M. A. Constructing inverse probability weights for marginal structural models. *Am. J. Epidemiol.* **2008,** *168*, 656-664.
2. Perkins, N. J.; Cole, S. R.; Harel, O.; Tchetgen Tchetgen, E. J.; Sun, B.; Mitchell, E. M.; Schisterman, E. F. Principled approaches to missing data in epidemiologic studies. *Am. J. Epidemiol.* **2018,** *187*, 568-575.
3. Ross, R. K.; Cole, S. R.; Edwards, J. K.; Westreich, D.; Daniels, J. L.; Stringer, J. S. A. Accounting for nonmonotone missing data using inverse probability weighting. *Stat. Med.* **2023,** *42*, 4282-4298.
4. Sun, B.; Perkins, N. J.; Cole, S. R.; Harel, O.; Mitchell, E. M.; Schisterman, E. F.; Tchetgen Tchetgen, E. J. Inverse-probability-weighted estimation for monotone and nonmonotone missing data. *Am. J. Epidemiol.* **2018,** *187*, 585-591.
5. Howe, C. J.; Cole, S. R.; Westreich, D. J.; Greenland, S.; Napravnik, S.; Eron, J. J., Jr. Splines for trend analysis and continuous confounder control. *Epidemiology* **2011,** *22*, 874-875.
6. Robins, J. M.; Hernan, M. A.; Brumback, B. Marginal structural models and causal inference in epidemiology. *Epidemiology* **2000,** *11*, 550-560.
7. Hernan, M. A.; Brumback, B.; Robins, J. M. Marginal structural models to estimate the causal effect of zidovudine on the survival of HIV-positive men. *Epidemiology* **2000,** *11*, 561-570.
